# A Case of Canine Polyglandular Deficiency Syndrome with Diabetes Mellitus and Hypoadrenocorticism

**DOI:** 10.3390/vetsci8030043

**Published:** 2021-03-07

**Authors:** Sho Furukawa, Natsuko Meguri, Kazue Koura, Hiroyuki Koura, Akira Matsuda

**Affiliations:** 1 Earth Animal Hospital, 4-3-43 Hokushin-cho, Kitami, Hokkaido 090-0052, Japan; furu.sho313@gmail.com (S.F.); natsukolovsummerktm@gmail.com (N.M.); kora@earth-pet.jp (H.K.); 2 Bihoro Animal Hospital, 51-8 Aoyamakita, Bihoro, Hokkaido 092-0066, Japan; ka.kora@earth-pet.jp; 3 Faculty of Veterinary Medicine, Okayama University of Science, 1-3 Ikoinooka, Imabari, Ehime 794-8555, Japan

**Keywords:** fludrocortisone, dog, polyglandular deficiency syndrome, diabetes mellitus, hypoadrenocorticism, desoxycorticosterone pivalate

## Abstract

This report describes the first clinical case, to our knowledge, of a dog with polyglandular deficiency syndrome with diabetes mellitus and hypoadrenocorticism. A six-year-old female Cavalier King Charles Spaniel presented with a history of lethargy and appetite loss. The dog was diagnosed with diabetic ketoacidosis based on hyperglycemia and renal glucose and ketone body loss. The dog’s condition improved on intensive treatment of diabetes mellitus; daily subcutaneous insulin detemir injection maintained an appropriate blood glucose level over half a year. However, the dog’s body weight gradually decreased from day 207, and on day 501, it presented with a decreased appetite; the precise cause could not be determined. Based on mild hyponatremia and hyperkalemia, hypoadrenocorticism was suggested; the diagnosis was made using an adrenocorticotropic hormone stimulation test. Daily fludrocortisone with low-dose prednisolone oral administration resulted in poor recovery of the blood chemistry abnormalities; however, monthly desoxycorticosterone pivalate (DOCP) subcutaneous injection with daily low-dose prednisolone oral administration helped in the significant recovery of the abnormalities. Therefore, clinicians should consider the possibility of coexistence of hypoadrenocorticism in dogs with diabetes mellitus presenting with undifferentiated weight loss. Additionally, DOCP (not fludrocortisone) may be useful in treating dogs with diabetes mellitus complicated with hypoadrenocorticism.

## 1. Introduction

Polyglandular deficiency syndrome (PDS), also described as autoimmune polyglandular syndrome (APS), is characterized by multiple endocrine end-organ failure [1,2]. In humans, this syndrome is known as autoimmune polyglandular syndrome (APS) and is categorized into four types, namely, type 1, type 2, type 3, and type 4 [3]. APS type 2 in humans is characterized by the occurrence of autoimmune hypoadrenocorticism with autoimmune thyroid diseases and/or type 1 diabetes mellitus. Human APS type 2 accounts for 34% of human primary hypoadrenocorticism, which is attributed to lymphoplasmacytic adrenalitis with autoimmune destruction of the adrenal cortex [3]. To date, although the combination of PDS and APS is clinically recognized frequently today, only a few cases of PDS with hypoadrenocorticism and hypothyroidism in canines have been reported in the literature [2,4,5,6]. However, to our knowledge, no case of PDS with hypoadrenocorticism and diabetes mellitus in dogs has yet been reported; the most favorable treatment remains unclear. We describe here a clinical case of canine PDS with diabetes mellitus and hypoadrenocorticism.

Primary hypoadrenocorticism is an uncommon disease in dogs [7]. Commonly, the zonae glomerulosa, fasciculata, and reticularis in the adrenal cortex are destroyed, resulting in a lack of both cortisol and aldosterone. Its etiology is not fully understood, but in some animals, an immune-mediated adrenal cortical destruction has been observed [8]. Most of the clinical signs, including lethargy, anorexia, decreased appetite, vomiting, and weight loss are intermittent in dogs with primary hypoadrenocorticism; however, some dogs present with hypovolemic shock with severe hyperkalemia, needing acute management with aggressive intravenous fluid resuscitation. Lifelong glucocorticoid and mineralocorticoid supplementation is warranted in chronic therapy for canine primary hypoadrenocorticism [7]. Either daily fludrocortisone oral administration or monthly desoxycorticosterone pivalate (DOCP) injection is used to supplement the mineralocorticoids. Fludrocortisone is a shorter-acting oral mineralocorticoid with some glucocorticoid activity, whereas DOCP has no glucocorticoid activity; thus, all dogs receiving DOCP require additional glucocorticoid administration [7]. Prednisolone is most frequently used for supplementing glucocorticoid at the physiological dose.

Diabetes mellitus is a common disorder characterized by hyperglycemia caused by defects in either insulin secretion or insulin sensitivity [9,10]. The diagnosis of diabetes mellitus is based on the presence of persistent hyperglycemia, glycosuria, and appropriate clinical signs, including polyurea, polydipsia, polyphagia, and weight loss. Most cases of diabetes mellitus in dogs resemble type 1 diabetes mellitus [9]. Many of the human patients with type 1 diabetes mellitus develop an autoimmune destruction of the pancreatic β-cells with autoantibodies; however, the etiology of diabetes mellitus in dogs also is not fully understood [9]. Some studies have reported that cell-mediated autoimmune destruction of β-cells was observed in up to 50% of dogs with diabetes; however, other studies found no evidence of autoimmune destruction [9,11,12]. Besides, diabetes mellitus in dogs is commonly characterized by an absolute requirement for exogenous insulin administration [10]. Sometimes insulin resistance is caused by hypercortisolism and an administration of glucocorticoids [9]. 

Herein, we describe a dog with diabetes mellitus accompanied by hypoadrenocorticism. The dog was treated with daily insulin injection and monthly DOCP injection, with daily oral administration of low-dose prednisolone.

## 2. Case History

On day 1, a six-year-old female Cavalier King Charles Spaniel, weighing 6.65 kg, presented with a history of lethargy, vomiting, appetite loss, polydipsia, and polyuria. The dog had lost 2.1 kg of its body weight in a month. On day 1, the blood chemistry revealed hyperglycemia, high blood urea nitrogen but normal creatinine, hypocalcemia, hyperphosphatasemia, hyperbilirubinemia, hyponatremia, hypokalemia, and hypochloremia (Table 1). Additionally, urine analysis revealed renal loss of glucose, ketone bodies, and protein. Diabetic ketoacidosis was diagnosed based on the initial evaluation. Fluid therapy with saline and intensive insulin therapy using a continuous infusion rate of regular insulin (0.1–0.5 IU/kg/h) was performed for the first three days. 

**Figure 1:**
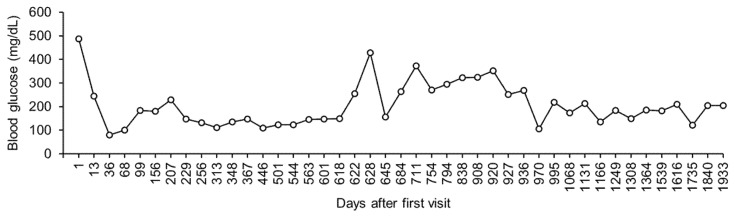
Change in blood glucose level. The blood glucose level was measured at 6 h following the subcutaneous administration of insulin detemir at least once every 10 days. Only the representative values are shown.

On day 4, there was no ketonuria, and insulin detemir was initiated to control the blood glucose levels (0.1–0.4 IU/kg, subcutaneous injection twice daily) On day 6, the dog’s appetite returned, and the dog was discharged. The dog owner started insulin injections at home. After day 6, the owner checked for glycosuria 2 to 6 times daily using urine test strips. The dog visited the hospital at least once every 10 days, where its glycemia and body weight were measured. In addition, blood urea nitrogen, creatinine, inorganic phosphorus, calcium, total protein, albumin, globulin, alanine aminotransferase, alkaline phosphatase, gamma glutamyl transferase, total bilirubin, and cholesterol were measured at least once a month. Figure 1 shows the representative values in blood glucose levels measured 6 h following the subcutaneous injection of insulin detemir.

**Figure 2:**
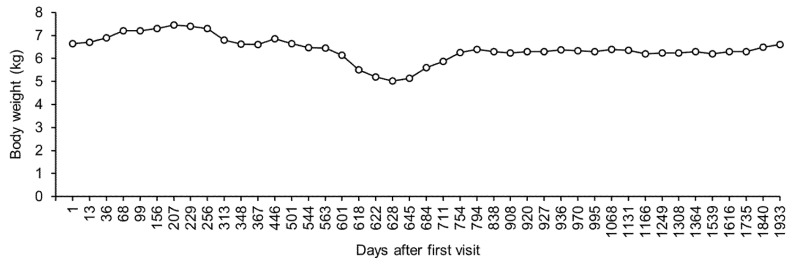
Change in body weight. Body weight was measured at least once every 10 days. Only the representative values are shown.

After day 207, the dog’s body weight gradually decreased and blood glucose was maintained at a relatively low level (Figure 2). However, the dog showed no clinical symptoms. On day 313, there was a marked decrease in the dog’s body weight, without an apparent appetite loss. Blood chemistry revealed no abnormal values, except slightly low inorganic phosphorus levels (Table 1). In addition, radiographic examination and ultrasonography revealed no abnormality in the dog. The owner complained that glycosuria was detected 1 to 2 times daily. To inject insulin more accurately, we asked the owner to use insulin detemir, which was diluted to 10 parts with saline. On day 348, the dog’s body weight decreased further. However, blood chemistry showed normal values, except for slightly low phosphorus levels (Table 1). The cause of the weight loss was unclear. Between day 348 and day 501, periodic blood chemistry monitoring was continued.

On day 501, the dog showed a decreased appetite and blood chemistry revealed hypoalbuminemia and hypocholesterolemia. The other values in blood chemistry showed normal values (Table 1). The determination of total thyroxin (TT4 = 0.9 μg/dL, reference range: 1.3–2.9 μg/dL), free thyroxin (FT4 = 2.1 μg/dL, reference range: 0.5–3.0 μg/dL), and thyrotropin (TSH = 0.04 μg/dL, reference range < 0.05 μg/dL) suggested the presence of euthyroid sick syndrome. The owner of the dog forced feeding to continue the injection of insulin. By day 508, the dog’s appetite had slightly improved. Between day 508 and day 544, although the dog’s appetite improved gradually, its body weight decreased further (Figure 2). On day 544, blood chemistry examination showed hypoproteinemia (Table 1). By day 563, the dog’s appetite and protein levels had returned to normal (Table 1). However, its weight decreased further, and there were no clinical symptoms detected between day 563 and day 618 (Figure 2). 

**Figure 3:**
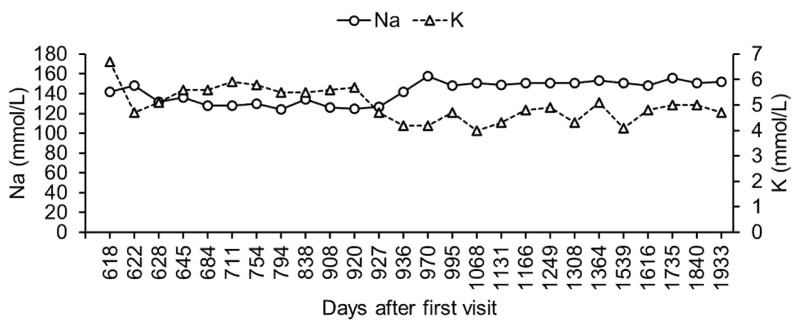
Changes in blood sodium and potassium level. Blood sodium and potassium were measured at least once every 10 days. Only the representative values are shown.

On day 618, the dog exhibited lethargy and appetite loss. Blood chemistry revealed hyperglycemia, high blood nitrogen, hypercreatinemia, hyperphosphatemia, hyponatremia, and hyperkalemia (Table 1). Sodium/potassium ratio was calculated as 21, suggesting the existence of adrenal insufficiency. The adrenocorticotropic hormone stimulation test showed undetected levels of serum cortisol before (reference range: 1.0–6.0 μg/dL) and after (reference range: 3.0–20 μg/dL) injection of adrenocorticotropic hormone intramuscularly. Therefore, the dog was diagnosed with hypoadrenocorticism. Fluid therapy with saline and oral fludrocortisone (9 μg/kg, twice daily) and prednisolone (0.1 mg/kg, twice daily) were administered. On day 622, the dog’s appetite was fully recovered, and the sodium/potassium ratio increased to 31 (sodium = 148 mmol/L; potassium = 4.7 mmol/L). Therefore, fluid therapy was stopped. By day 628, the sodium/potassium ratio had decreased to 24 (sodium = 136 mmol/L; potassium = 5.6 mmol/L). Despite the fluctuation in the sodium/potassium ratio, the dog had a good clinical condition. Between day 628 and day 711, the dog’s appetite was stable, and its body weight started to increase gradually (Figure 2). However, there was persistent hyponatremia and hyperkalemia (Figure 3). 

On day 711, the sodium/potassium ratio decreased to 22 (sodium = 128 mmol/L; potassium = 5.9 mmol/L), and the dog was asymptomatic. The dose of fludrocortisone (13 μg/kg, twice a day) was increased, to help normalize the sodium/potassium ratio. However, hyponatremia and hyperkalemia did not improve by day 920 (Figure 3). Additionally, the blood glucose levels remained high (Figure 1). 

By day 920, although the dog had a good general condition, hyponatremia and hyperkalemia persisted (sodium = 125 mmol/L; potassium = 5.7 mmol/L). We then stopped the oral administration of fludrocortisone and initiated a subcutaneous injection of DOCP (2.2 mg/kg, once per 25–30 days) and the oral administration of prednisolone (0.1 mg/kg, twice a day). By day 927, blood potassium levels became very low (4.7 mmol/L). By day 936, further hyponatremia and hyperkalemia persisted (sodium = 142 mmol/L; potassium = 4.2 mmol/L). 

Between day 936 and day 1933, the dog visited the hospital, where a periodic monitoring of its body weight and its blood glucose, sodium, and potassium levels was performed. During this period, there was no hyponatremia or hyperkalemia (Figure 3). In addition, both the body weight and blood glucose levels were well controlled (Figure 1). 

By day 1933, the dog was still treated with subcutaneous injections of insulin and DOCP, and oral administration of prednisolone. The dog’s general state was good, without any abnormal increase or decrease in blood glucose, sodium, and potassium levels.

## 3. Discussion

In most dogs with PDS, each endocrinopathy typically appears separately; hence, the diagnosis of supplementary disorders is delayed. The diagnosis of hypoadrenocorticism was reportedly delayed by 3–4 months following the diagnosis of hypothyroidism in two dogs with PDS [2,6]. In our case, the second disorder manifested more than a year following the first diagnosis of diabetes mellitus. Moreover, following the appearance of decreased appetite, diagnosis could take approximately four months. Hypoalbuminemia, hypocholesterolemia, and a relatively low blood glucose level may be important clues for early diagnosis. To our knowledge, the coexistence of diabetes mellitus and hypoadrenocorticism in dogs has not been reported previously. In humans, APS type 2 with hypoadrenocorticism and type 1 diabetes mellitus rarely occurs [3]. Although it is unknown whether PDS in dogs has a pathogenesis similar to that of APS in humans, lymphocytic adenohypophysitis and adrenalitis have been reported in dogs with hypothyroidism and hypoadrenocorticism [5]. While there was minimal or no clinical evidence for an autoimmune disease etiology in our case, the dog in this study could be diagnosed with PDS based on the occurrence of two endocrinopathies. When there is an unidentified decrease in body weight or loss of appetite during the treatment of diabetes mellitus in dogs, appropriate examination should be performed, considering the complications associated with hypoadrenocorticism and other coexisting disorders.

In our case, oral administration of fludrocortisone had a poor effect on hyponatremia and hyperkalemia, whereas DOCP proved to be effective. Studies have reported that 8.9% of dogs with primary hypoadrenocorticism treated with fludrocortisone either showed a poor response or experienced side effects [13]. In addition, DOCP was reported to have stronger mineralocorticoid effects than fludrocortisone [14]. During the transcription of the study, DOCP was commercially unavailable in Japan; however, the use of DOCP should be considered in cases resistant to fludrocortisone. In our case, an increased blood glucose level was observed during the administration of fludrocortisone. Fludrocortisone, in addition to mineralocorticoid activity, has glucocorticoid activity [7]. An increased blood glucose level may suggest increased insulin resistance caused by the glucocorticoid activity of fludrocortisone. 

In this study, we reported the case of a dog with PDS accompanied by diabetes mellitus and hypoadrenocorticism. Our case suggests that the administration of DOCP, not fludrocortisone, with insulin and low-dose prednisolone, may be considered the first choice of treatment for cases of PDS with diabetes mellitus and hypoadrenocorticism in dogs.

## Figures and Tables

**Table 1:** Representative results of blood chemistry.

Item	Day 1	Day 313	Day 348	Day 501	Day 544	Day 563	Day 618	Reference Range
Glucose (mg/dL)	488	143	136	124	124	146	204	60–123
Blood urea nitrogen (mg/dL)	34	7	9	13	27	23	31	9–30
Creatinine (mg/dL)	0.6	0.6	0.6	0.7	0.6	0.6	1.7	0.5–1.4
Inorganic phosphorus (mg/dL)	4	2.2	3.1	3.1	3.4	4.1	8.9	2.2–5.9
Calcium (mg/dL)	7.2	9.4	10.8	9.6	9.0	9.3	10.2	9.0–11.4
Total protein (g/dL)	6.7	6.5	6.4	5.8	5.0	5.6	6.2	5.1–7.5
Albumin (g/dL)	3.3	2.8	3.2	2.2	2.4	2.7	2.9	2.6–3.9
Globulin (g/dL)	3.4	3.7	3.2	3.6	2.6	2.9	3.3	2.1–4.3
Alanine aminotransferase (IU/L)	69	50	22	21	27	28	43	18–93
Alkaline phosphatase (IU/L)	404	142	191	63	56	55	60	15–162
Gamma glutamyl transferase (IU/L)	4	0	0	0	0	5	5	0–9
Total bilirubin (mg/dL)	1	0.1	0.4	< 0.1	< 0.1	0.1	0.3	0–0.4
Cholesterol (mg/dL)	323	203	226	61	116	81	77	132–344
Sodium (mmol/L)	120	-	-	149	-		142	141–156
Potassium (mmol/L)	3.6	-	-	4.4	-		6.7	3.9–5.5
Chloride (mmol/L)	85	-	-	115	-		107	109–121

## Data Availability

The data presented in this study are available on request from the corresponding author.
